# Advances in the Chemistry and Biology of Specialised Pro-Resolving Mediators (SPMs) †

**DOI:** 10.3390/molecules29102233

**Published:** 2024-05-10

**Authors:** Lucy Byrne, Patrick J. Guiry

**Affiliations:** Centre for Synthesis and Chemical Biology, UCD School of Chemistry, University College Dublin, Belfield, D04 N2E5 Dublin, Ireland

**Keywords:** inflammation, lipoxin, resolvin

## Abstract

This review article assembles key recent advances in the synthetic chemistry and biology of specialised pro-resolving mediators (SPMs). The major medicinal chemistry developments in the design, synthesis and biological evaluation of synthetic SPM analogues of lipoxins and resolvins have been discussed. These include variations in the top and bottom chains, as well as changes to the triene core, of lipoxins, all changes intended to enhance the metabolic stability whilst retaining or improving biological activity. Similar chemical modifications of resolvins are also discussed. The biological evaluation of these synthetic SPMs is also described in some detail. Original investigations into the biological activity of endogenous SPMs led to the pairing of these ligands with the FPR2/LX receptor, and these results have been challenged in more recent work, leading to conflicting results and views, which are again discussed.

## 1. Introduction

In ancient times, the process of inflammation was thought to be an undesirable action and harmful to the host. However, since the 19th century, the inflammatory response has been recognised as part of the healing process and key to the defensive mechanisms of the body [1]. Inflammation is part of the innate immune response to infection or injury, leading to a series of physiological responses in the host. These responses are characterised by heat, redness and pain, often accompanied by swelling and a loss of function. Collectively, they represent the five ‘cardinal signs’ of inflammation [2].

The aim of the inflammatory response is to confine the insult to an isolated area and to initiate the immune response to repair the injured tissue and regenerate tissue homeostasis. This is achieved by the release of chemical mediators such as cytokines and chemokines, which activate the physical response, increasing vasodilation and vascular permeability in order to flood the infected area with immune cells [3]. In heathy cells, when the injury is eradicated, the inflammatory response resolves, and subsequent immune reactions diminish. The resolution phase is essential to the return of tissue homeostasis [4]. The breakdown of this aspect of the inflammatory response leads to a prolonged exposure to pro-inflammatory mediators, leading to chronic inflammatory diseases such as cardiovascular disease, arthritis, asthma and diabetes [5].

Once the inflammatory response is initiated, the loop of inflammatory events occurs in a cascade until the infection is eradicated by the immune response or the injury is contained. Early actions of the host response are taken over by more complex mechanisms and eventually become redundant. It is pivotal that the inflammatory response is controlled and resolved. Many molecules play a role in governing the duration and magnitude of the inflammatory response. Lipoxins are one such class of anti-inflammatory, pro-resolving molecules [6]. These endogenous, lipid-derived, chemical mediators are a class of eicosanoid natural products. They are part of a cohort of molecules, collectively referred to as specialised pro-resolving mediators (SMPs), that play an active role in the resolution phase of inflammation. The purpose of this review is to summarise recent advances in the chemistry and biology of these specialised pro-resolving mediators, with a focus on lipoxins and resolvins.

## 2. Lipoxins

**Figure 1:**
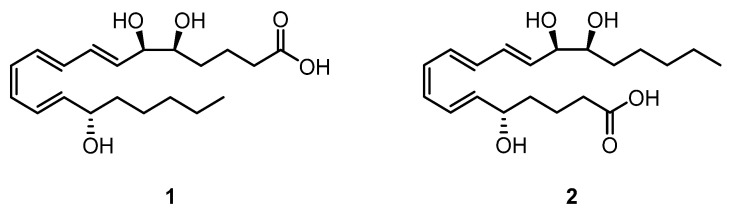
Native LXA_4_ (**1**) and LXB_4_ (**2**).

Lipoxins, and other members of this class of molecules, evoke their effect by inhibiting the congregation of neutrophils to the site of infection, thus triggering the cascade of events that culminate in the resolution of the inflammatory response [7]. They are also known to promote wound healing by promoting the infiltration of monocytes, which are required for this purpose. As well as the endogenous lipoxin molecules, LXA_4_ (**1**) and LXB_4_ (**2**), other specialised pro-resolving mediators, such as resolvins, protectins and maresins, also play an important role in the resolution of inflammation (Figure 1) [8].

**Scheme 1:**
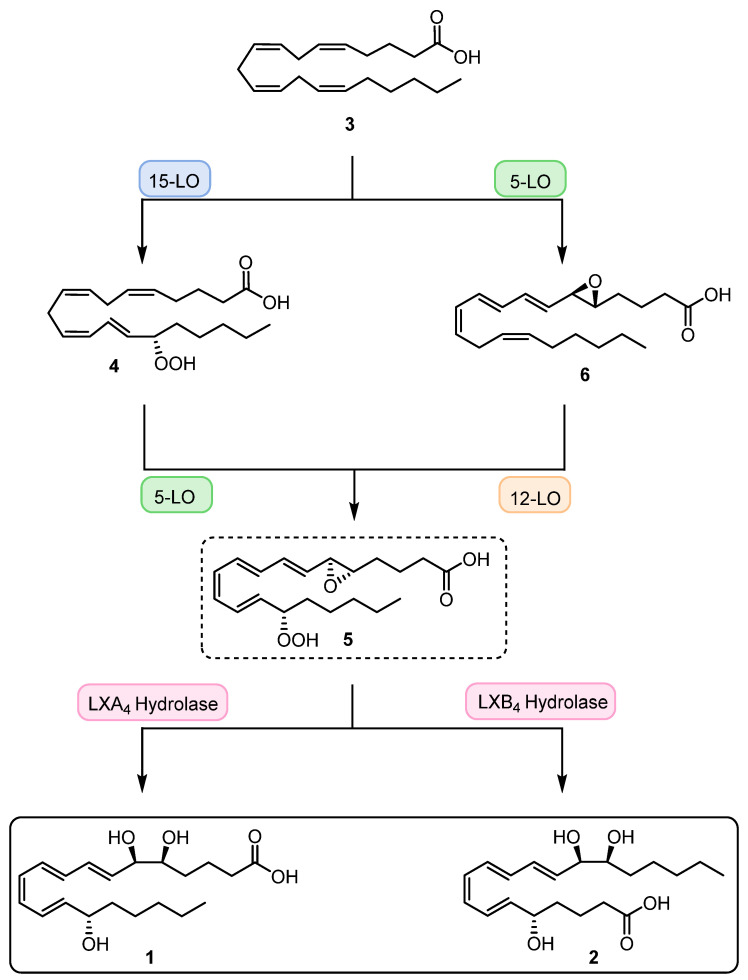
Biosynthetic pathway to endogenous LXA_4_ (**1**) and LXB_4_ (**2**).

Lipoxins were first isolated from human leukocytes in 1984 by Serhan, Hamberg and Samuelsson [9]. Their continued investigations on the subject allowed the precise biosynthesis of the naturally occurring lipoxins LXA_4_ and LXB_4_ to be understood [10]. Lipoxins are a derivative of the arachidonic acid pathway which is known to play a key role in the process of inflammation. As such, it has been the subject of considerable academic interest. Non-esterified arachidonic acid is subject to oxidation by both the lipoxygenase and the cyclooxygenase pathways. The three major lipoxygenase pathways, 5-, 12- and 15-lipoxygenase, transform arachidonic acid into its biological active derivatives (Scheme 1). These enzymes stereospecifically insert molecular oxygen into the unconjugated double bond system [10].

One of the major biosynthetic routes to LXA_4_ (**1**) and LXB_4_ (**2**) involves the insertion of molecular oxygen at the carbon-15 position of arachidonic acid (**3**), which is facilitated by the action of the enzyme 15-lipoxygenase (15-LO). This oxidation of arachidonic acid forms 15-hydroperoxyeicosatetraenoic acid (15-HPETE) (**4**), which is subject to further oxidation by 5-lipoxygenase to form an epoxide intermediate (**5**). This molecule is rapidly hydrolysed to form LXA_4_ and LXB_4_ by an attack on the C-6 position or the C-14 position, respectively [10].

A second LX biosynthesis pathway, involving the interaction of human neutrophils with platelets in the blood, was discovered by Serhan and co-workers [11]. In this pathway, arachidonic acid is subject to oxidation by 5-LO to form LTA_4_ (**6**). Platelet 12-LO acts on **6** to form the epoxide intermediate **5**, which is subsequently transformed into LXA_4_ (**1**) by action at the C-6 position and LXB_4_ (**2**) by action at the C-14 position by the corresponding LX hydrolase enzymes.

## 3. Aspirin-Triggered Lipoxins

**Figure 2:**
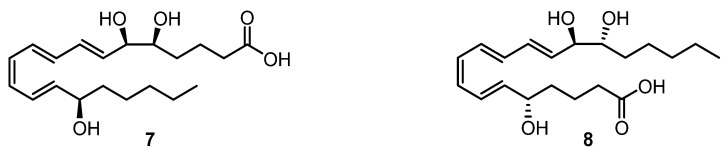
Aspirin-triggered lipoxins 15-*epi*-LXA_4_ (**7**) and 15-*epi*-LXB_4_ (**8**).

Aspirin also plays a key role in the biosynthesis of lipoxins, as reported by Claria and Serhan in 1995 [12]. The aspirin dependent route leads to the formation of 15-*epi*-LXA_4_ (**7**), also known as aspirin-triggered lipoxin (ATL). The aspirin-triggered pathway can also lead to the formation of the LXB_4_ mimetic (**8**) (Figure 2).

Aspirin is a well-known pharmaceutical agent with a broad scope of clinical applications. It is commonly prescribed as an analgesic, antipyretic, cardiovascular and anti-cancer treatment [13].

**Scheme 2:**
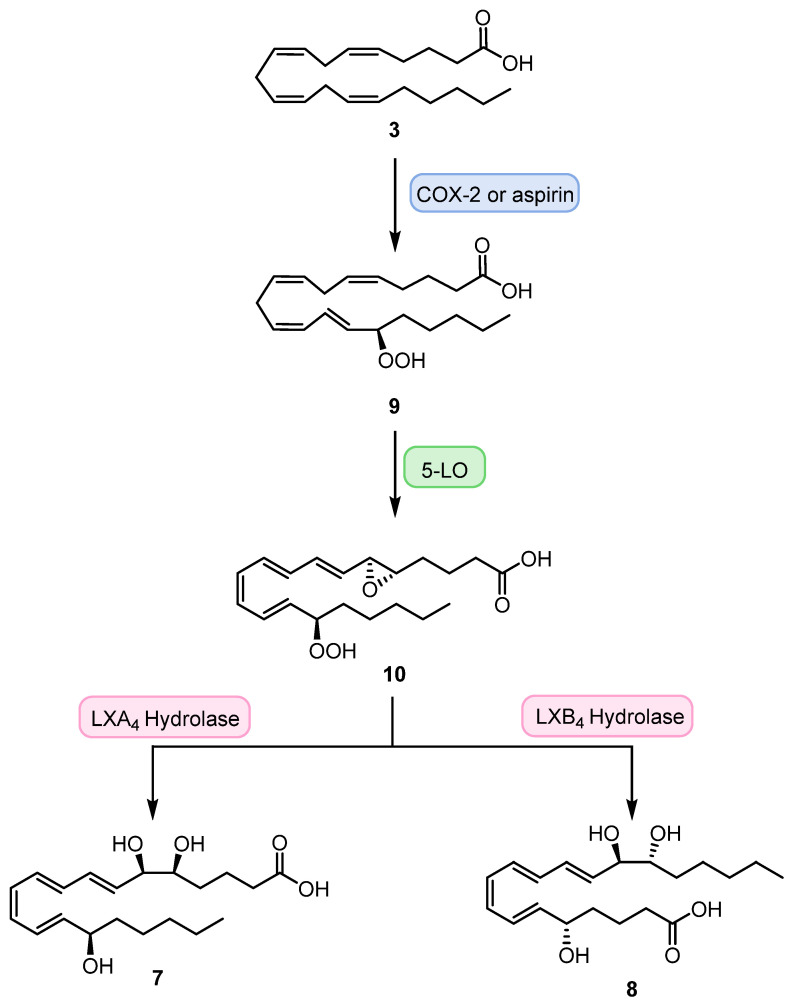
Biosynthesis of ATLs 15-epi-LXA_4_ (**7**) and 15-epi-LXB_4_ (**8**).

Cyclooxygenase-2 (COX-2) is an enzyme that acts on arachidonic acid **3** to form prostaglandins in the inflammatory reaction cascade [14]. COX-2 switches its catalytic activity in the presence of aspirin, generating 15-hydroxyperoxyeicosatetraenoic acid (**9**) (15-HPETE) instead of prostaglandins (Scheme 2). When acetylated by aspirin, COX-2 enzymatically converts arachidonic acid to 15-HPETE. *Epi*-lipoxins are formed by the metabolism of 15-HPETE (**9**) by the 5-LO enzyme. The synthesis first goes through a 5(*S*)-epoxytetraene intermediate (**10**). This intermediate leads to the formation of both 15-*epi*-LXA_4_ (**7**) and 15-epi-LXB_4_ (**8**), which carry the (*R*)-configuration at C-15 [15]. Numerous studies have proven that ATLs can serve as endogenous anti-inflammatory signals and facilitate some of aspirin’s beneficial actions [16].

## 4. Biological Activity of LXA_4_

Studies conducted by Serhan and colleagues have demonstrated the potent anti-inflammatory and pro-resolving effects of LX and ATL, both in vitro and in vivo. The primary target of these eicosanoids are neutrophils, which express high levels of certain G-protein coupled receptors. Lipoxins and their derivatives bind to the G-protein coupled lipoxin A_4_ receptor (ALX), originally named due to its high affinity for LXA_4_. This receptor is currently identified as FPR2/ALX, reflecting agonism by both formylated peptides and LXA_4_, and is consistent with the Nomenclature and Standards Committee of the International Union of Basic and Clinical Pharmacology singles receptor nomenclature guidelines [17].

The binding of LX to this receptor triggers the release of chemo signals through which LX exert their anti-inflammatory effects. LXA_4_ is known to bind in a stereoselective fashion to its receptor, which is not shared by either LXB_4_ or LTB_4_ [18].

The ALX receptor was identified through binding studies using tritium-labelled LXA_4_ (^3^H-LXA_4_) [19]. Functional LXA_4_ receptors are inducible in HL-60 cells. Serhan and co-workers were therefore able to test orphan cDNAs, cloned from these HL-60 cells, which encode 7-transmembrane region receptors, for their ability to bind and signal with LXA_4_. Chinese hamster ovary (CHO) cells, transfected with the orphan receptor cDNA, displayed specific tritium-labelled LXA_4_ high-affinity binding. Bioactive ATL and LXA_4_ analogues compete with ^3^H-LXA_4_ binding to LXA_4_ receptors, thereby confirming their binding action [19].

After binding to these receptors, LXs show counterregulatory effects in some tissues. They inhibit the movement of polymorphonuclear leukocytes (PMNs), impeding their adhesion to endothelial cells [20]. This inhibits the chemotaxis of PMNs and eosinophils. They also produce vasodilatory effects and inhibit leukotriene-B_4_-mediated inflammatory events [21]. One of the primary anti-inflammatory effects of lipoxins is that they evoke a series of bio-actions that inhibit neutrophil infiltration to the injury site, thus preventing tissue injury [22]. After neutrophils are cleared from the site of injury/infection, macrophages are recruited. Macrophages are cells that remove dead and pathogenic cells from the site of infection. Lipoxins promote the resolution of inflammation by delaying the apoptosis of macrophages [23]. The biological importance of lipoxins in the inflammatory cascade is evidenced by the fact that a deficiency of these compounds in the body is associated with human inflammatory diseases, including asthma, glomerulonephritis and rheumatoid arthritis [24].

## 5. Metabolic Stability of Native Lipoxins

**Figure 3:**
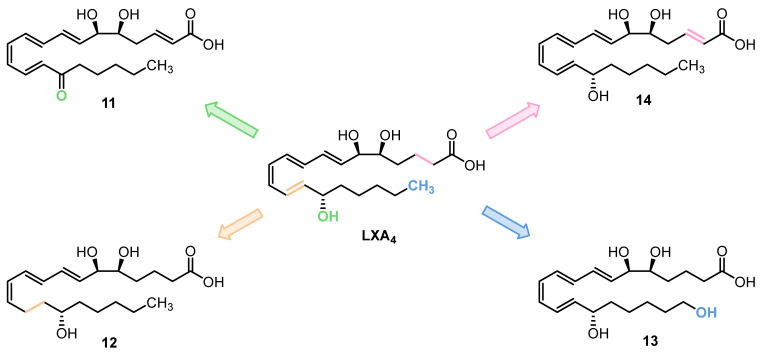
Pathways of the enzymatic degradation of LXA_4_.

While native lipoxins are capable of promoting the resolution of acute inflammation, the full spectrum of their therapeutic application is hindered by the inherent chemical lability of the conjugated tetraene, which makes up a part of their molecular skeleton. The native endogenous mediators are rapidly metabolised in vivo. This is a trait characteristic of all autocoids. The inactivation of lipoxins is achieved through enzymatic degradation soon after they carry out their anti-inflammatory effects. The rapid metabolism of native lipoxins has been well studied, and many of the pathways are known (Figure 3).

Lipoxins undergo oxidation at the C-15 position, mediated by the action of 15-hydroxyprostaglandin dehydrogenase (15-PGDH), which results in their inactivation. 15-PGDH catalyses the dehydrogenation reaction of the C-15 hydroxyl to afford the corresponding ketone, 15-oxo-LXA_4_ (**11**) [25]. A reaction will also occur at the C13-C14 double bond, the reduction of which by the enzyme leukotriene B_4_ 12-hydroxydehydrogenase (PGR/LTB_4_DH) will result in the formation of the 13,14-dihydro-LXA_4_ metabolite. LTB_4_DH will also react with the ketone (**11**) to form 13,14-dihydro-15-oxo-LXA_4_ (**12**) [26]. Another known pathway of LX metabolism is the ω-oxidation at the C-20 position to form **13**. This is mediated by cytochrome P450 (believed to be CYP3A) enzymes. Lipoxins are also subject to metabolism via the β-oxidation/elimination pathway, which delivers the α,β-unsaturated metabolite (**14**) [27]. These metabolites display a considerable reduction in activity compared to the native lipoxin.

## 6. Development of Stable Lipoxin Analogues

Although native lipoxins display potent anti-inflammatory properties, they are not considered to be viable candidates as therapeutic agents, due primarily to their poor half-life in vivo. Although the transient nature of lipoxins is a significant drawback regarding their therapeutic application, the potential benefit that could be drawn from these native anti-inflammatory molecules is indisputable.

**Figure 4:**
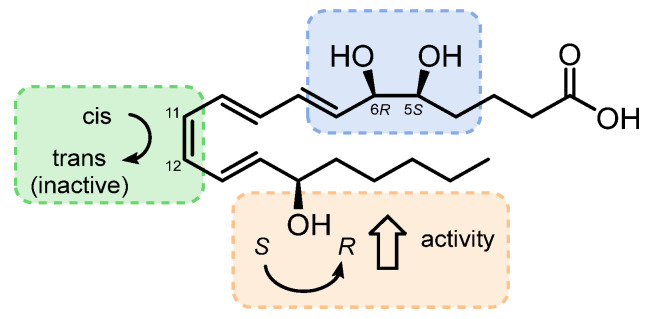
SAR of native LXA_4_.

Significant work was undertaken regarding the Structure–Activity Relationship of the native LXA_4_, which determined the features of the molecule that were crucial to its biological activity [22]. The results of these experiments determined that for appropriate binding to the ALX receptor, the 5(*S*)-6(*R*)-configurations of the upper chain diol and the *cis*-configuration of the C-11 alkene were necessary (Figure 4). Additionally, the 15-*epi*-LXA_4_, which is the aspirin-triggered variant and possesses opposite stereochemistry at the C-15 alcohol, was determined to have greater activity compared to the native LXA_4_ (Figure 4) [22].

**Figure 5:**
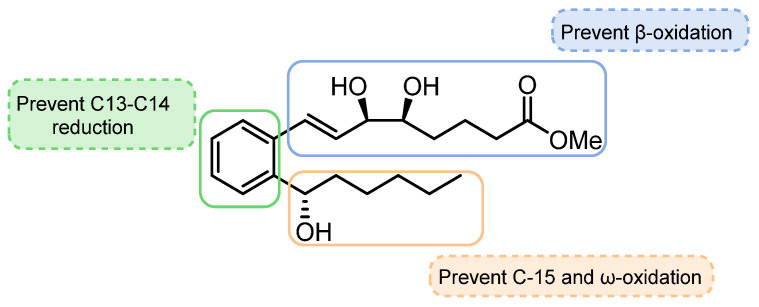
Rational design of LXA_4_ analogues.

Working with these observations, and also considering the mode of the enzymatic inactivation of the native LXA_4_, investigations began into the synthesis of analogues of LXA_4_ and LXB_4_ that possess similar biological action to that of their native counterparts but possess a resistance to metabolic inactivation. With the pathways of lipoxin metabolism identified, analogues could be specifically designed to circumvent these undesirable reactions (Figure 5) [28].

## 7. LXA_4_ Triene Core Modifications

**Figure 6:**
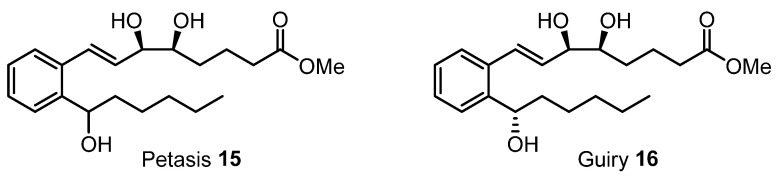
Benzo-LXA_4_ analogues.

The reduction of the C13-C14 double bond majorly contributes to the deactivation of the native lipoxin. Our group and others have devoted significant work to the modification of the triene core to avoid this eventuality. The first compounds of this kind were the benzo-containing analogues. Benzo-LXA_4_ was synthesised by Petasis et al. (**15**) in 2008 and asymmetrically by Guiry (**16**) in 2007 (Figure 6) [29,30].

This analogue is resistant to reduction at the C13-C14 position, and the benzo-modification improves their half-life substantially. Benzo-LXA_4_ is equally as efficient as native LXA_4_ but displays a 1000-fold increase in potency, demonstrating anti-inflammatory activity in acute models of hind limb ischemia–reperfusion injury and zymosan-induced peritonitis, including reduced PMN infiltration and pro-inflammatory cytokine release [30,31].

The therapeutic efficacy of this series of mimetics was further established in additional experimental models, including surgically induced renal fibrosis [32], obesity-driven adipose inflammation [33] and diabetes-induced atherosclerosis and kidney disease [34,35].

Several pathological features were observed in a high-fat diet-induced murine obesity model, including fatty liver, impaired glucose tolerance, adipose inflammation and CKD, and benzo-LXA_4_ was found to mimic many of the protective effects of LXA_4_. Benzo-LXA_4_ was also found to restore the expression of the autophagy markers LC3-II and p62, which were reduced by obesity [33].

In collaboration with Godson, the therapeutic potential of benzo-LXA_4_ was investigated, comparing it to LXA_4_ in an established model of diabetic complications (streptozotocin-treated ApoE^−/−^ mice). Benzo-LXA_4_ was found to protect against diabetes-induced vascular complications in a similar manner to native LXA_4_, albeit at a lower dose [34,35]. Importantly, our study demonstrated that LXA_4_ and benzo-LXA_4_ suppress fibrotic gene expression by negatively regulating the early growth response factor (EGR-1) transcription factor network, a complex dysregulated in human renal fibrosis [35].

In the investigations of diabetes-induced atherosclerosis and diabetic kidney disease, LXA_4_ and benzo-LXA_4_ were used in two different dosing modalities: as disease developed along a 10- or 20-week time course of diabetes or as a treatment of established disease. LXA_4_ and benzo-LXA_4_ prevented the development of vascular complications of diabetes and, most importantly, reversed established disease.

Several key aspects of disease pathology were affected, including the inhibition of glomerular matrix accumulation, proteinuria [35] and the regression of atheromatous plaque [34]. In exploring the underlying mechanisms, it was shown that LXs inhibited monocyte adhesion to endothelia, vascular smooth muscle cell proliferation and inflammatory responses, including NF-κB-driven gene expression and TNF-α release. The latter responses were mirrored in human plaque tissue cultured ex vivo and exposed to LXs [34]. Importantly, the LX-induced attenuation of vascular complications was independent of changes in diabetes-induced hyperglycemia and elevated glycosylated hemoglobin A1c.

**Figure 7:**
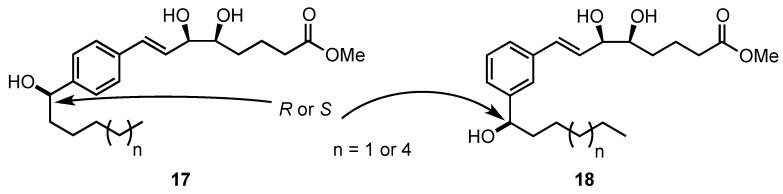
1,4- and 1,3-substituted benzo-LXA_4_ analogues.

As an extension of the benzo-LXA_4_ work, we synthesised a set of eight novel Lipoxin A_4_ analogues (Figure 7), in which we varied the substitution pattern around the benzene ring to afford analogues mimicking potential conformers of the native LXA_4_ that may adapt in vivo [36]. We achieved an enantioselective synthesis of these analogues with both the native C15 configuration as well as the epi-configuration of the aspirin-triggered LXA_4_. We also extended the alkyl chain length in four of the analogues to observe the effect on bioactivity. Our preliminary results demonstrate the anti-inflammatory potential of these novel LXA_4_ analogues. The tests showed that the longer lower-chain analogues demonstrated better biological activity than the shorter-chain analogues with the 1,4-disubstituted analogue **17** (n = 4) and 1,3-disubstituted analogue **18** (n = 4), showing a significant reduction in the production of specific pro-inflammatory cytokines, IL-12p40 and IL-1β, at low concentrations.

**Figure 8:**
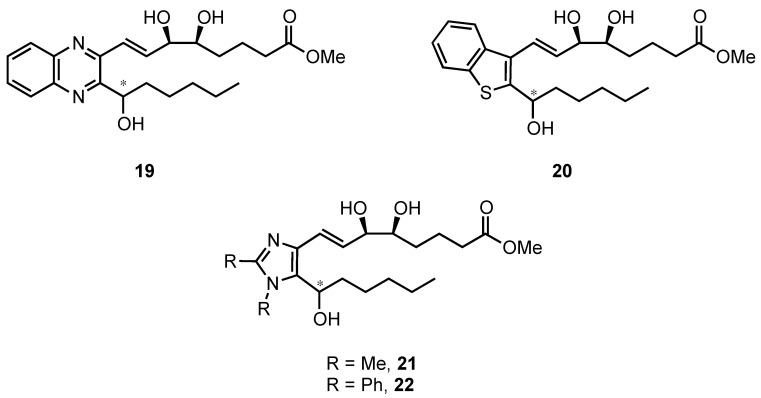
Range of heteroaromatic LXA_4_ analogues (* indicates a chiral centre and both epimers were prepared).

Following the generation of the benzo-LXA_4_ analogues, the scope of this concept was expanded to include a range of aromatic and heteroaromatic-substituted Lipoxin analogues by Guiry. Nitrogen-containing heterocycles such as quinoxaline (**19**) and benzothiophene (**20**) were incorporated [37,38]. More recently, dimethyl-(**21**) and di-phenyl-imidazole (**22**) were synthesised and evaluated for their biological activity (Figure 8). It was hoped that these modifications to the triene core of the molecular skeleton would imbue the analogue with a resistance to metabolic inactivation and thereby increase its activity.

Eight novel quinoxaline-containing analogues, in which the length of the lower alkyl chain was varied, were screened for their impact on inflammatory responses [37]. Structure–activity relationship (SAR) studies showed that (*R*)-**19** was the most efficacious and potent anti-inflammatory compound of those tested, as it significantly attenuated lipopolysaccharide (LPS)- and tumor-necrosis-factor-α (TNF-α)-induced NF-κB activity in monocytes and vascular smooth muscle cells. The NF-κB family of transcription factors is activated by several pro-inflammatory mediators [39]. The molecular target of (*R*)-**19** was investigated, and it was determined to activate the endogenous LX receptor formyl peptide receptor 2 (ALX/FPR2). The anti-inflammatory properties of (*R*)-**19** were further investigated in vivo in murine models of acute inflammation. Consistent with in vitro observations, (*R*)-**19** attenuated inflammatory responses, and these results support the therapeutic potential of the lead QNX-sLXm (*R*)-**19** in the context of novel inflammatory regulators.

In 2023, Guiry reported the asymmetric synthesis of lipoxin A_4_ (LXA_4_) mimetics, in which the triene core of the molecule has been replaced by an aromatic sulfur-containing benzothiophene ring (**20**) [40]. The key steps in the synthesis included a Friedel–Crafts acylation, a Suzuki coupling between two upper and lower chain fragments and a highly stereoselective Noyori transfer hydrogenation to set the stereochemistry of the alcohol at the benzylic position. No biological evaluation of this analogue was reported.

Subsequent testing of the anti-inflammatory properties of the imidazole-containing analogues (**21**–**22**) showed that the native LXA_4_ is capable of reducing LPS-induced NF-κB activity by 24 ± 1% at a 100 nM concentration. However, the dimethylimidazole (**24**) was found to reduce the anti-inflammatory activity by a greater margin of 44 ± 9% and at a reduced concentration of 1 pM and was therefore identified as the lead compound, showing the greatest potency and efficiency of any analogue synthesised to date [38].

## 8. LXA_4_ Lower Chain Modifications

**Figure 9:**
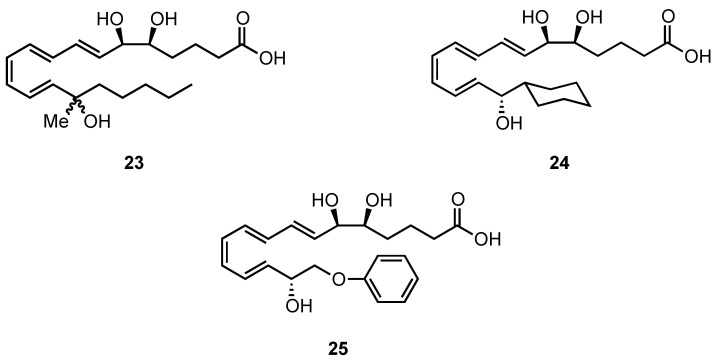
Lower-chain modified LXA_4_ analogues.

Early work on this front came from Petasis and co-workers, who designed a number of LXA_4_ analogues containing variations at the C-15 and C-20, in 1995 [41]. They published lower-chain modified mimetics such as 15-(*R*/*S*)-methyl LXA_4_ (**23**), 15-cyclohexyl-LXA_4_ (**24**) and 16-phenoxy-LXA_4_ (**25**) (Figure 9). These analogues were designed to hinder the metabolic deactivation through C-15-oxidation and to evaluate the importance of the C-15 alcohol. To test the reactivity of these new lipoxin mimetics, each molecule was subjected to a system of PMA differentiated HL-60 cells, a system which rapidly metabolises the native LXA_4_. Studies conducted by Petasis and co-workers showed that after exposure to this system for a period of 15 min, less than 10% of LXA_4_ can be recovered. Conversely, the new analogues **23**, **24** and **25** were recoverable, after an incubation of 2 h, in up to a 95% yield. In addition to displaying increased longevity, these C-15-modified mimetics also retained the biological action of LXA_4_ by successfully inhibiting the transmission of PMNs.

**Figure 10:**
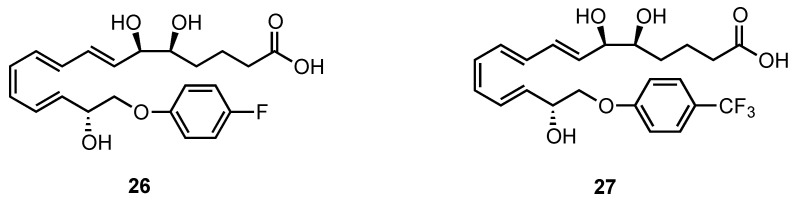
*para*-Fluorophenoxy (**26**) and *para*-trifluoromethyl (**27**) LXA_4_ analogues.

In an effort to overcome ω-oxidation, *para*-fluoro-phenoxy (**26**) and *para*-trifluoromethylphenyl LXA_4_ (**27**) were later designed and prepared (Figure 10) [42]. These analogues also displayed increased biostability and reactivity when compared to the native lipoxin.

**Figure 11:**
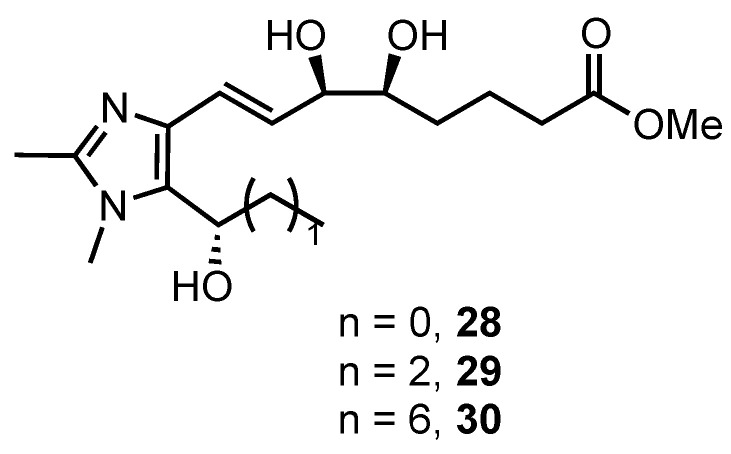
Dimethyl-LXA_4_ with alkyl chain length variation.

Lower-chain modifications were also carried out within the Guiry group. After the imidazole-containing analogue was identified as a lead compound of the heteroaromatic-containing series, further studies on this mimetic were also carried out by varying the length of the lower alkyl chain in analogues **28**, **29** and **30** (Figure 11) [38]. The imidazole-containing analogues possessing truncated alkyl chains displayed a reduction in potency compared to the native lower-chain-containing analogue.

**Figure 12:**
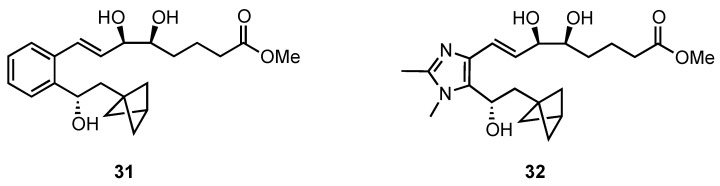
Bicyclo[1.1.1]pentane LXA_4_ mimetics **31** and **32**.

LXA_4_ mimetics designed to withstand the reduction of the C2 alkene to the C3 alkene and the ω-oxidation of C20 were synthesised by Guiry in 2022 (Figure 12) [43]. These mimetics, **31** and **32**, featured a bicyclo[1.1.1]pentane ring as part of the bottom chain of their structure. This functionality was incorporated to block ω-oxidation, increasing the stability of the mimetics, which increases their biological activity. Analogue **32** was found to be more potent and efficacious in inhibiting NF-kB-induced luciferase gene expression than native LXA_4_ and **26**, demonstrating 50% inhibition in the picomolar range. These results indicate that the presence of the bicyclo[1.1.1]pentane ring in the bottom chain of the mimetic increases the activity of the mimetic.

## 9. Upper-Chain Modifications

**Figure 13:**
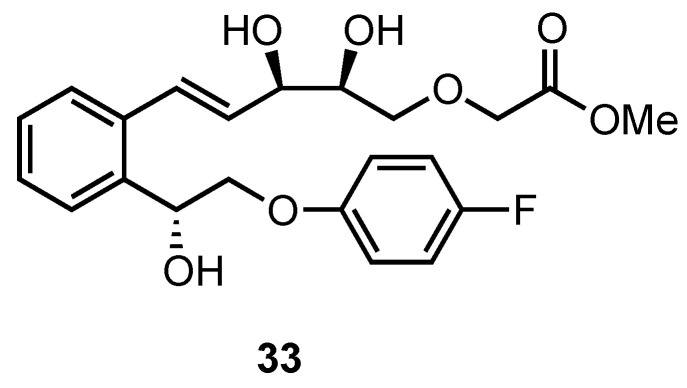
3-oxo-*para*-fluorophenoxy LXA_4_ (**33**).

Although extensive research has been conducted into the variation in the aromatic region and the lower chain of LXA_4_, changes to the upper chain remain largely unexplored. In fact, only one analogue possessing a modified upper chain has been presented in the literature (Figure 13). The previously described *para*-fluorophenoxy LXA_4_ mimetic (**26**) was further altered by Serhan and colleagues in 2004 [44]. This 3-oxo-derivative (**33**) displayed similar efficacy and potency to those of the corresponding native-upper-chain analogue. However, the newly modified upper chain did exhibit increased chemical and metabolic stability.

## 10. Resolvins

Lipoxins are only one family of the polyunsaturated fatty acid (PUFA) metabolites, termed specialised pro-resolving mediators (SPMs), that regulate the inflammatory system. This genus of endogenous lipid mediators includes lipoxins, resolvins, protectins and, more recently, maresins [45].

**Figure 14:**
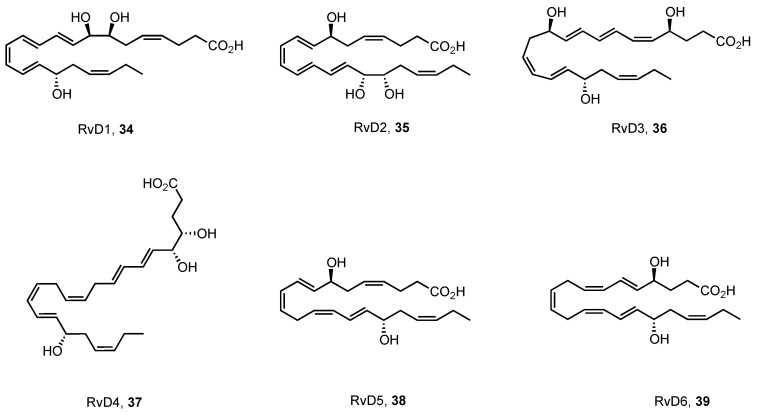
Resolvin D-series.

Resolvins, derived from ‘resolution phase interaction products’, are autocoids biosynthesised from essential PUFAs. There are two subcategories of resolvin molecules: the E-series resolvins, which are derived from eicosapentaenoic acid (EPA), and the D-series resolvins, which are derived from docosahexanoic acid (DHA) [46]. There are six known compounds in the D-series, commonly referred to as RvDs (Figure 14).

Of these resolvin D-series, two compounds, RvD1 (**34**) and RvD2 (**35**), are trihydroxylated and contain a tetraene core. The presence of these functionalities lends the molecules a structural resemblance to lipoxin-type mimetics. Inversely, they possess structural qualities distinct from lipoxins, namely, *cis*-unsaturation on both the upper and lower chains and an elongated upper chain by two carbon units. RvD3 (**36**) and RvD4 (**37**) are also trihydroxylated DHA-derivatives, whereas RvD5 (**38**) and RvD6 (**39**) possess only two alcohol groups. Similar to the lipoxin class, aspirin-triggered resolvins have also been identified, with the inversion of stereochemistry at the lower-chain alcohol [47]. Of the compounds classed as SPMs, these resolvins bear the closest resemblance to the lipoxin family—particularly, RvD1 and RvD2, which exhibit structural parallels to LXA_4_ and LXB_4_, respectively. RvD1 had also demonstrated a higher natural resistance to metabolic inactivation than LXA_4_, making it a compound worthy of further investigation [47].

## 11. RvD1 in Inflammation

Many chemical mediators, such as the arachidonic acid-derived lipid mediators, have well-established roles in the process of inflammation. Extensive research into the subject eventually demonstrated that arachidonic acid was not the only fatty acid precursor that undergoes transformation to produce bioactive mediators in inflammation and resolution. The ω-3 fatty acid-derived EPA and DHA also deliver a number of such molecules [48]. RvD1, which is derived from DHA, is now known to produce anti-inflammatory action but also to promote resolution back to the non-inflamed state [49]. The resolution phase of inflammation was once believed to be a passive process; however, the discovery of these resolvin-type molecules, as endogenous stop-signals for inflammation, has provided the evidence required to identify resolution as an active and regulated process [50].

RvD1 is produced in resolving exudates in vivo, as a product of the transcellular biosynthesis with human leukocyte and endothelial cells [47]. It was also identified in human whole blood and in the murine brain [51]. The compound is biosynthesised by the sequential oxidation of DHA by 15-LOX and 5-LOX [47].

The anti-inflammatory properties of RvD1 were investigated using a murine skin air pouch model, which examined the effect of RvD1 on PMN accumulation [52]. Polymorphonuclear neutrophils (PMNs) contribute to the immune response by recruiting various proinflammatory mediators, which, in healthy tissues, would promote wound-healing. In the case of inflammatory autoimmune diseases, the presence of PMNs causes an exacerbation of debilitating disease symptoms. The study, conducted by Serhan in 2002, demonstrated the ability of RvD1 to regulate PMN action in vivo [52]. The local administration of RvD1 also inhibited zymosan-induced peritonis by the regulation of leukocyte recruitment into the air pouch.

A later study in 2006 showed that RvD1 has a suppressive effect on the release of the proinflammatory cytokine TNF-α, which is implicated in the pathogenesis of ischemic acute kidney injury. This work highlighted the active role of RvD1 in the resolution of inflammation. Not only do they block the activation of proinflammatory pathways, but they also act during the acute injury phase to counteract inflammation and injury [53].

A study conducted in 2011 discovered the protective action of resolvin compounds in corneal angiogenesis [54]. The injury occurs in this case through cysteinyl leukotrienes, which stimulate conjunctival goblet cell mucous secretion. This is a crucial component of ocular allergy, usually treated by the sustained use of steroids. However, RvD1 and RvE1 have been shown to block the action of cysteinyl leukotrienes by preventing the increase in Ca^2+^ and thereby preventing the activation of the ERK1/2 receptor. Resolvins have been recorded to inhibit physiological processes of inflammation, decrease neutrophil assembly and block cytokine production. Based on these actions, resolvins have reduced inflammation in animal models of several cases of chronic inflammatory diseases [53,55].

Another aspect of RvD1 that makes it an attractive target for further studies is the receptor upon which it acts, as it is capable of activating the FPR2/ALX receptor [56]. This is exceedingly advantageous in a strategic sense, as it mitigates the need to develop a new testing method for synthetic lipoxin–resolvin analogues. Receptor selectivity studies demonstrate that the pro-resolving actions of RvD1 and AT-RvD1 are mediated by two G-protein coupled receptors, FPR2/ALX and GPR32 [57]. The receptor of interest in this circumstance is the FPR2/ALX receptor, as it is used in the biological testing of the synthetic LXA_4_ analogues prepared by Guiry. The FPR2/ALX receptor is activated only by the presence of LXA_4,_ RvD1, RvD3 and their aspirin-triggered counterparts.

## 12. Synthetic Analogues of RvD1

**Figure 15:**
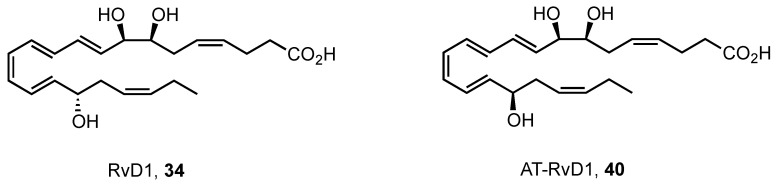
RvD1 and AT-RvD1.

To date, the investigation into the synthetic analogues of the resolvin family has been relatively limited in comparison to that of its lipoxin counterparts. The first total synthesis of RvD1 and its aspirin-triggered C-17 epimer, AT-RvD1 (**40**), was reported in 2007 (Figure 15) [47].

**Figure 16:**
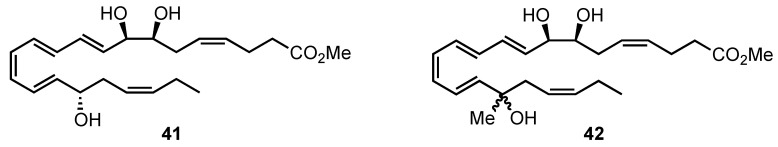
RvD1 methyl ester **41** and 17-methyl RvD1 methyl ester **42**.

In 2008, Petasis presented the RvD1 methyl ester (**41**) and the 17-methyl RvD1 methyl ester (**42**), representing the first synthetic mimetics of this class (Figure 16) [58]. Analogue **42** has yet to be prepared stereoselectively, although this molecule did demonstrate some promise in biological testing, where it displayed organ-protective functions.

**Figure 17:**
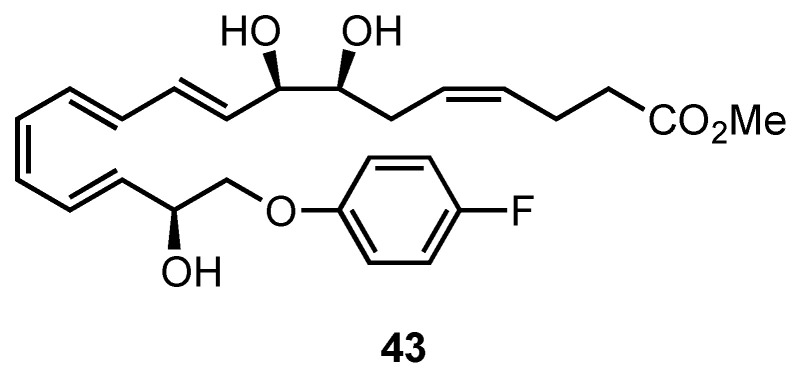
17*R*-19-*para*-Fluorophenoxy-RvD1 (*p*-RvD1) **43**.

17*R*-19-*para*-Fluorophenoxy-RvD1 (*p*-RvD1) (**43**) was then reported, also by Serhan, Petasis and co-workers (Figure 17). This molecule was tested for protective function in inflammatory lung injury, an important model for tissue injury. The inflammation stems from the deposition of IgG immune complexes in the lung tissue. The biological assay was completed with *p*-RvD1 and AT-RvD1, and its conclusion indicated that both AT-RvD1 and *p*-RvD1 successfully suppressed the inflammatory response induced by IgG immune complexes, both in vivo and in vitro [59].

**Figure 18:**
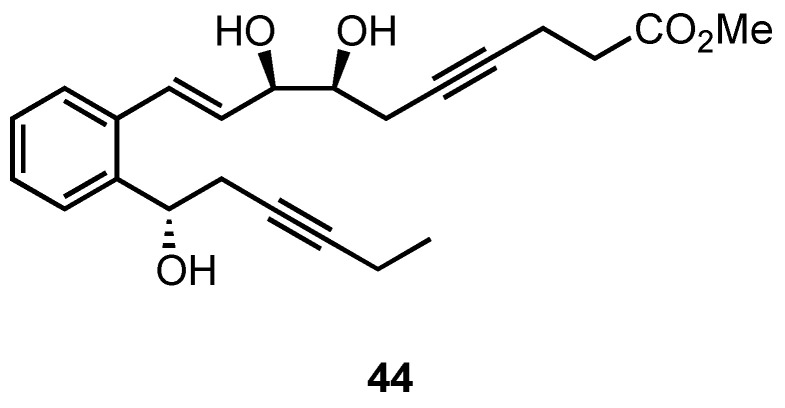
Benzo-diacetylenic-17-*R*-RvD1 methyl ester (**44**).

The protective function displayed by RvD1 inspired the desire for the construction of a more stable analogue, thus leading to the synthesis of benzo-diacetylenic-17-*R*-RvD1 methyl ester (**44**) (Figure 18) [60]. This more stable mimetic is an agonist of the human GPR32 receptor. An in vivo mouse model displayed its ability to suppress inflammation by enhancing the phagocytosis of macrophage cells. In testing, the new analogue **44** was found to be equipotent with RvD1 in accelerating the resolution of inflammation. The presence of the ring stabilises the backbone of the native RvD1, instilling a resistance to degradation that could prove useful in the design of future therapeutics [60].

## 13. Synthetic Analogues of RvE1

**Figure 19:**
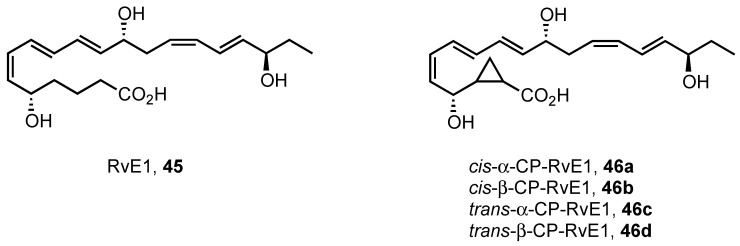
RvD1 (**45**) and cyclopropane congeners (**46a**–**d**).

Resolvin E1 (RvE1, **45**), one of the four resolvin E-series, is an ω-3 fatty acid eicosapentaenoic acid (EPA) metabolite with very potent anti-inflammatory activity identified by Serhan (Figure 19) [61,62]. Its remarkable anti-inflammatory effects are due to the inhibition of neutrophil chemotaxis and inflammatory cytokine production and the promotion of macrophage phagocytosis. Resolvins are widely studied, yet only a few analogues of RvE1 are reported. On the basis of a conformational analysis of RvE1, Shuto and Fukoda designed its four cyclopropane congeners (**46a**–**d**), in which the conformationally flexible terminal C1–C4 moiety of RvE1 was rigidified by introducing stereoisomeric cyclopropanes [63]. The four congeners, along with RvE1, were efficiently synthesised using a common synthetic route. The evaluation of the anti-inflammatory effects of the compounds in mice resulted in the identification of *trans*-β-CP-RvE1 (**46d**), which was significantly more active than RvE1, as a potential lead for anti-inflammatory drugs of a novel mechanism of action.

## 14. Conflicting Views on the Detection of SPMs and the Identification of Proposed GPCR

This review collates recent advances in terms of the design and synthesis of specialised pro-resolving mediators that have been inspired by the chemical structures of the naturally occurring lipoxins and resolvins. One hallmark of SPM formation in vivo is that the reported levels of these lipid mediators are much lower than typical pro-inflammatory mediators including leukotrienes or certain cyclooxygenase-derived prostaglandins. Thus, the reliable detection and quantification of these metabolites are challenging, even employing HPLC-MS instrumentation with low limits of detection. For a balanced perspective in the current review, it is appropriate to note that there is some disagreement, if not controversy, about the identity and the signalling of the proposed G-protein-coupled SPM receptors, thus challenging the role of SPMs as endogenous mediators of the resolution of inflammation, as noted in the recent review by Schebb and Steinhilber [64]. They suggest that evidence that lipoxins and resolvins exert their effects through specific receptors remains controversial and incomplete. Finally, evidence that SPMs are formed in biologically active concentrations in humans that promote the resolution of inflammation has been questioned.

One example from the primary literature that questions that activation of human and murine forms of FPR2/ALX by LXA_4_ and analogues was systematically examined by Offermanns and co-workers [65]. They showed that both receptor orthologues responded to the FPR2/ALX peptide agonist WKYMVM when expressed heterologously. In contrast, LXA_4_ from different sources neither increased [Ca^2+^]_i_ and extracellular-signal-regulated kinase (ERK) phosphorylation nor induced a decrease in cAMP levels or a translocation of β-arrestin. Also, several LXA_4_ analogues were found to be unable to signal through FPR2/ALX. They concluded that FPR2/ALX is not activated by LXA_4_ and that the molecular mechanism by which LXA_4_ functions still needs to be identified. A further example comes from the report from Riddy, who comprehensively showed how several natural mediators and synthetic ligands signal through three specialised pro-resolving mediator GPCRs using multiple ligands from different classes across four-six endpoint signalling assays. Their study discovers new ligand pairings, refutes others, reveals poly-pharmacology and identifies biased agonism in FPR2/LXA_4_ receptor pharmacology [66].

## 15. Conclusions

In conclusion, this review article reports a concise collation of recent advances in the synthetic chemistry and biology of specialised pro-resolving mediators (SPMs). The major medicinal chemistry developments in the design and synthesis of synthetic SPM analogues of lipoxins and resolvins have been discussed. Original investigations discovering the biological activity of SPMs led to the pairing of these ligands with the FPR2/LX receptor, and these results have been challenged in more recent work, leading to conflicting results and views. Irrespective of this, there is an ongoing effort to provide novel therapeutic agents to combat an array of inflammatory diseases, and it is hoped that this timely review will help to stimulate the design and biological evaluation of novel lipoxin and resolvin analogues.

## Data Availability

The data supporting the results discussed in this review can be found in the primary literature.
